# Supplementary material for: Novel Driver Strength Index highlights important cancer genes in TCGA PanCanAtlas patients
Source: PeerJ. 2022 Aug 11;10:e13860. doi: 10.7717/peerj.13860 (PMC9375969; doi:10.7717/peerj.13860)
Supplement: Supplemental Information 2 [file peerj-10-13860-s002.zip › COHORTS/genes plots/cohorts/2021_8_16_14_9_distribution_cohorts_HNSC.pdf]

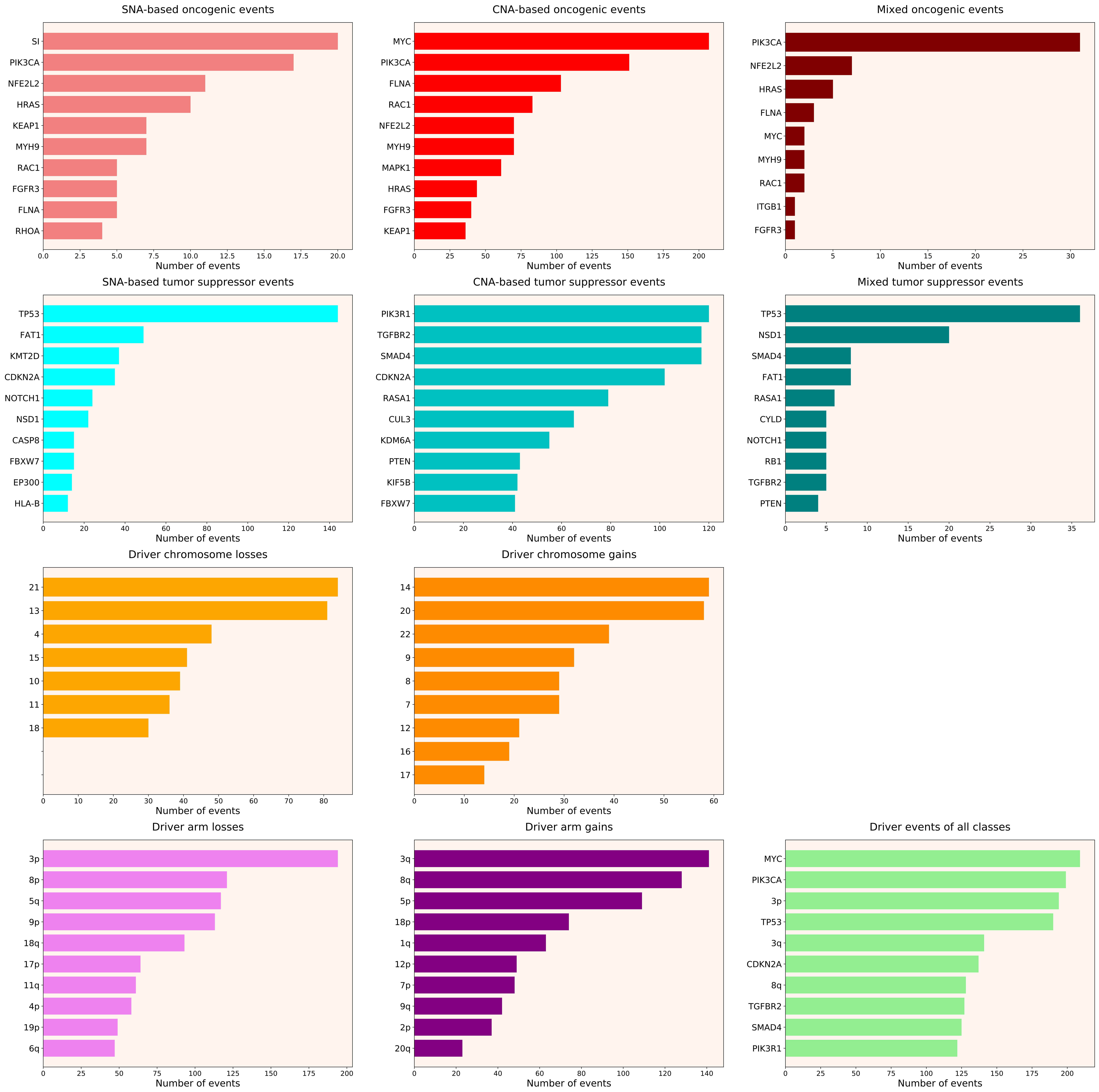
SNA-based oncogenic events
SI
PIK3CA
NFE2L2
HRAS
KEAP1
MYH9
RAC1
FGFR3
FLNA
RHOA
0.0 2.5 5.0 7.5 10.0 12.5 15.0 17.5 20.0
Number of events
CNA-based oncogenic events
MYC
PIK3CA
FLNA
RAC1
NFE2L2
MYH9
MAPK1
HRAS
FGFR3
KEAP1
0 25 50 75 100 125 150 175 200
Number of events
Mixed oncogenic events
PIK3CA
NFE2L2
HRAS
FLNA
MYC
MYH9
RAC1
ITGB1
FGFR3
0 5 10 15 20 25 30
Number of events
SNA-based tumor suppressor events
TP53
FAT1
KMT2D
CDKN2A
NOTCH1
NSD1
CASP8
FBXW7
EP300
HLA-B
0 20 40 60 80 100 120 140
Number of events
CNA-based tumor suppressor events
PIK3R1
TGFBR2
SMAD4
CDKN2A
RASA1
CUL3
KDM6A
PTEN
KIF5B
FBXW7
0 20 40 60 80 100 120
Number of events
Mixed tumor suppressor events
TP53
NSD1
SMAD4
FAT1
RASA1
CYLD
NOTCH1
RB1
TGFBR2
PTEN
0 5 10 15 20 25 30 35
Number of events
Driver chromosome losses
21
13
4
15
10
11
18
0 10 20 30 40 50 60 70 80
Number of events
Driver chromosome gains
14
20
22
9
8
7
12
16
17
0 10 20 30 40 50 60
Number of events
Driver arm losses
3p
8p
5q
9p
18q
17p
11q
4p
19p
6q
0 25 50 75 100 125 150 175 200
Number of events
Driver arm gains
3q
8q
5p
18p
1q
12p
7p
9q
2p
20q
0 20 40 60 80 100 120 140
Number of events
Driver events of all classes
MYC
PIK3CA
3p
TP53
3q
CDKN2A
8q
TGFBR2
SMAD4
PIK3R1
0 25 50 75 100 125 150 175 200
Number of events
